# Noninvasive *in vivo* magnetic resonance measures of glutathione synthesis in human and rat liver as an oxidative stress biomarker

**DOI:** 10.1002/hep.26925

**Published:** 2014-04-25

**Authors:** John T Skamarauskas, Fiona Oakley, Fiona E Smith, Carlo Bawn, Michael Dunn, Daniel S Vidler, Matthew Clemence, Peter G Blain, Roy Taylor, Michael P Gamcsik, Peter E Thelwall

**Affiliations:** 1 Institute of Cellular Medicine, Newcastle UniversityNewcastle upon Tyne, UK; 2 Newcastle Magnetic Resonance Center, Newcastle UniversityNewcastle upon Tyne, UK; 3 Northern Institute for Cancer Research, Newcastle UniversityNewcastle upon Tyne, UK; 4 Medical Toxicology Center, Newcastle UniversityNewcastle upon Tyne, UK; 5 Philips Healthcare Clinical ScienceGuildford, UK; 6 Joint Department of Biomedical Engineering, University of North Carolina, Chapel Hill and North Carolina State UniversityRaleigh, NC

## Abstract

Oxidative stress (OS) plays a central role in the progression of liver disease and in damage to liver by toxic xenobiotics. We have developed methods for noninvasive assessment of hepatic OS defenses by measuring flux through the glutathione (GSH) synthesis pathway. ^13^C-labeled GSH is endogenously produced and detected by *in vivo* magnetic resonance after administration of [2-^13^C]-glycine. We report on a successful first-ever human demonstration of this approach as well as preclinical studies demonstrating perturbed GSH metabolism in models of acute and chronic OS. Human studies employed oral administration of [2-^13^C]-glycine and ^13^C spectroscopy on a 3T clinical magnetic resonance (MR) imaging scanner and demonstrated detection and quantification of endogenously produced ^13^C-GSH after labeled glycine ingestion. Plasma analysis demonstrated that glycine ^13^C fractional enrichment achieved steady state during the 6-hour ingestion period. Mean rate of synthesis of hepatic ^13^C-labeled GSH was 0.32 ± 0.18 mmole/kg/hour. Preclinical models of acute OS and nonalcoholic steatohepatitis (NASH) comprised CCl_4_-treated and high-fat, high-carbohydrate diet-fed Sprague-Dawley rats, respectively, using intravenous administration of [2-^13^C]-glycine and observation of ^13^C-label metabolism on a 7T preclinical MR system. Preclinical studies demonstrated a 54% elevation of GSH content and a 31% increase in flux through the GSH synthesis pathway at 12 hours after acute insult caused by CCl_4_ administration, as well as a 23% decrease in GSH content and evidence of early steatohepatitis in the model of NASH. *Conclusion*: Our data demonstrate *in vivo* ^13^C-labeling and detection of GSH as a biomarker of tissue OS defenses, detecting chronic and acute OS insults. The methods are applicable to clinical research studies of hepatic OS in disease states over time as well as monitoring effects of therapeutic interventions.

Tissue oxidative stress (OS) results from an excess of reactive oxygen species (ROS), resulting in an inability of cells to maintain a reduced intracellular environment. Hepatic OS is central to the processes involved in progression of liver disease,^1^–^3^ and the cell has evolved multiple mechanisms to protect itself from OS, including DNA repair, enzymes that catalyze the deactivation of free radicals, and antioxidants of endogenous or dietary origin. ROS and pro-oxidants can arise within a tissue from multiple endogenous and exogenous sources, such as by-products of oxidative phosphorylation, components of an immune response, from xenobiotics, or the generation of reactive intermediates as a result of catabolic processes.^1^ The principal hepatic intracellular antioxidant is glutathione (GSH), a tripeptide of glycine, cysteine, and glutamate that is synthesized in two steps from its constituent amino acids by γ-glutamylcysteine synthetase and GSH synthetase.^3^,^4^ GSH has a central role in protecting the cell against ROS, and in detoxifying xenobiotics. Homeostatic processes strive to maintain hepatic GSH content and maintain a reduced intracellular environment.

A wealth of preclinical studies have investigated hepatic responses to OS by change in GSH content or GSH synthesis enzyme activities.^5^ In addition, blood-based indirect markers of OS have been identified, typically comprising by-products of OS (e.g., lipid peroxidation [LPO] by-products),^6^–^8^ along with measurements in liver biopsy samples of GSH content, enzyme activities, and expression of genes related to OS defense, as reported on previously.^9^,^10^ However, direct tissue sampling is invasive and carries an associated risk to the patient, and blood biomarkers report on downstream effects of OS rather than providing direct measures of cellular redox defenses.

We have developed methods to monitor liver GSH metabolism *in vivo*, providing a noninvasive biomarker that reports directly on hepatic OS defences. In our previous studies, we have demonstrated that administration of ^13^C-labeled glycine results in the metabolic production of γ-glutamylcysteinyl-[2-^13^C]-glycine (referred to hereafter as ^13^C-GSH), and that *in vivo* monitoring of this ^13^C-labeling process can be performed by magnetic resonance imaging (MRI).^11^–^13^ In this study, we translate this approach to the human liver for clinical research studies and show alterations in GSH metabolism in response to chronic and acute OS insults in preclinical models.

Our preclinical studies employed two models of hepatic OS: an acute insult resultant from CCl_4_ administration and chronic OS generated from a high-fat, high-carbohydrate (HFHC) diet model of steatohepatitis. CCl_4_ administration has been widely employed to generate hepatic OS and study hepatotoxicity, fibrosis, hepatocellular death, and carcinogenicity^14^ and allows us to gauge change in hepatic GSH turnover after acute OS insult. Our diet-induced steatohepatitis model was based on that described by Kohli et al.^15^ as a model of chronic OS, which exhibited a nonalcoholic steatohepatitis (NASH) phenotype. Our model comprised an 8-week diet duration, with the aims of producing early NASH and testing whether our magnetic resonance (MR) approach can detect changes in tissue OS defenses at this stage of NASH progression.

The aim of our study was to translate the GSH ^13^C-labeling approach to human studies, to perform preclinical studies of controlled acute and chronic OS insult, and thus demonstrate the utility of GSH metabolism monitoring as a biomarker of perturbed hepatic redox defences suitable for application to clinical research studies.

## Materials and Methods

### Preclinical Study: Experimental Design

Preclinical studies were performed under a project license granted by the Home Office in accord with the Animals (Scientific Procedures) Act of 1986. [2-^13^C]-Glycine was purchased from Cambridge Isotope Laboratories, Inc. (Andover, MA) and from Sigma-Aldrich Co. Ltd. (Gillingham, UK). Three groups of male Sprague-Dawley rats (Charles River UK, Ltd., Margate, UK) comprised control, CCl_4_-induced acute OS, and rats fed an HFHC diet to induce steatohepatitis (n = 7 per group). CCl_4_-treated rats received a 1:1 mixture of CCl_4_ and refined olive oil (0.1 mL per 100 g body weight, intraperitoneal [IP] injection) at 12 hours before the MR study. The HFHC diet comprised *ad libitum* feed containing 60% kcal from fat (TestDiet 58R2; IPS Ltd., London, UK) and drinking water supplemented with 55 mmol/L of sucrose and 128 mmol/L of fructose for 8 weeks before MR experiments.

### Preclinical Study: Magnetic Resonance Spectroscopy

**Fig 1:**
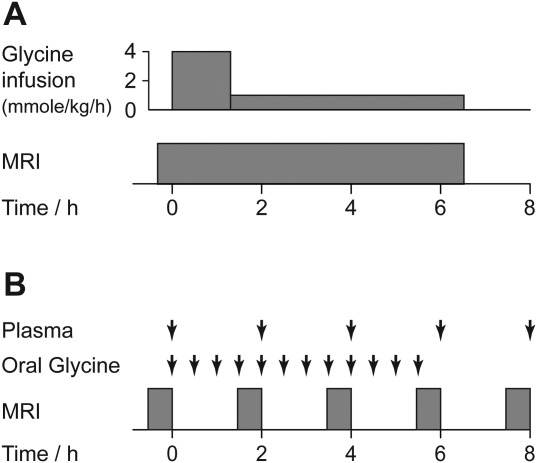
MR study protocol summary. (A) Preclinical study protocol showing timings of glycine infusion and MRI scanning. (B) Human study protocol showing timings of blood sampling, oral glycine administration, and MRI scanning.

**Fig 2:**
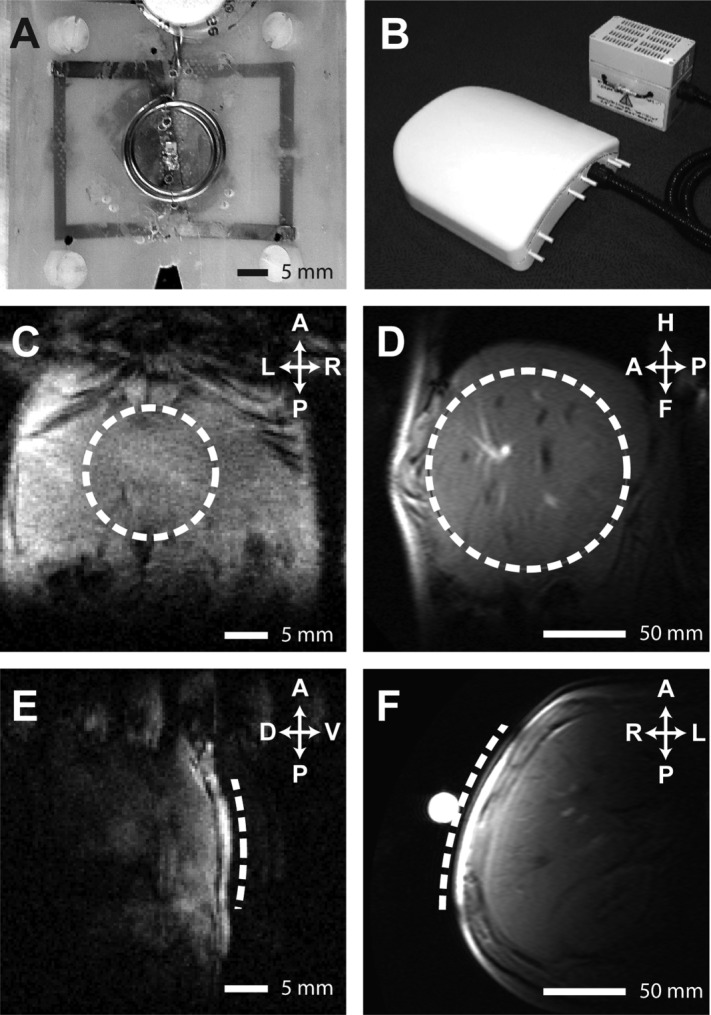
^13^C/^1^H RF coils, and MR images showing coil positioning. (A and B) ^13^C/^1^H RF coils employed for preclinical and human studies, respectively. (C-F) Representative hepatic ^1^H images showing location of ^13^C surface coils (dashed line) for preclinical (C and E) and human (D and F) studies.

Figure 1A shows the relative timing of [2-^13^C]-glycine infusion and MR data acquisitions. MR studies were started at the same time each day (10 a.m.) to avoid the influence of diurnal variation in GSH synthesis rate on study data.^16^ Rats were anesthetized with IP-administered solution of fentanyl (0.79 mg/mL), fluanisone (2.5 mg/mL), and midazolam (1.25 mg/mL) in water at a dose of 0.2 mL/kg, and maintenance doses were administered as required through an MR-compatible IP cannula. Intravenous (IV) administration of [2-^13^C]-glycine in water (1 M, pH 7.4) was through a tail vein catheter. Respiratory and body temperature monitoring was performed during MR experiments. ^1^H-decoupled ^13^C spectra were acquired on a Varian 7T magnet and spectrometer (DirectDrive system; Varian, Inc., Palo Alto, CA) using a custom ^13^C/^1^H coil (15 mm diameter ^13^C coil; Fig. 2A). Positioning of the coil relative to the liver is shown in Fig. 2C,E. ^13^C spectra were acquired every 10 minutes for the experiment duration (tip angle = 90 degrees; repetition time [TR] = 1.5 seconds; spectral width [SW] = 10 kHz; 400 averages). [2-^13^C]-glycine delivery commenced after acquisition of baseline MR data at a loading dose of 4 mmoles/kg/hour for 90 minutes, then at a maintenance dose of 1 mmole/kg/hour for 5 hours. Details of preparation of liver tissue perchloric acid extracts, of proton nuclear magnetic resonance (^1^H NMR) and mass spectrometry (MS) analysis of liver and plasma samples, preparation of histological section, and MR data analysis and quantitation are provided in the Supporting Materials. Statistical significance of differences in concentrations and rates between experimental groups was determined using one-way analysis of variance with Tukey's multiple comparisons (Minitab 16; Minitab Inc., State College, PA).

### Human Study: Subjects and Experimental Design

Ethical permission for the study was obtained from the Newcastle and North Tyneside 1 Research Ethics Committee. Healthy male volunteers (n = 3) were recruited from the staff of Newcastle University (Newcastle upon Tyne, UK) and informed consent was obtained. Subjects abstained from alcohol for 3 days before the study and fasted from 11.30 p.m. on the evening before the study (water *ad libitum*). The study commenced at 8.30 a.m. the following day with acquisition of baseline liver ^13^C spectra. Figure 1B shows timing of [2-^13^C]-glycine administration, blood sampling, and MR examinations. [2-^13^C]-glycine was purchased from Cambridge Isotope Laboratories, administration consisted of 12 doses of 3 g of [2-^13^C]-glycine dissolved in 50 mL of water, ingested orally at 30-minute intervals over a 6-hour period. Glycine administration commenced immediately after acquisition of baseline liver MR spectra and MR data acquisition and was repeated at 2, 4, 6, and 8 hours. Plasma samples were stored at −40°C before analysis of total and [2-^13^C]-labeled glycine content by ^1^H NMR, as described in the Supporting Materials. Volunteers ate a standardized lunch (comprising a sandwich, an apple, and water) at 165 minutes after commencing glycine ingestion. The experimental protocol was designed to replicate the preclinical studies as closely as possible, though [2-^13^C]-glycine administration was oral instead of by IV infusion. This provided delivery of label to the liver through the portal vein and represented a pragmatic approach for ethical review committee consideration of this first-ever healthy volunteer study.

### Human Study: ^13^C Magnetic Resonance Spectroscopy

MRI and spectroscopy (MRS) measurements were made using a Philips Achieva 3T whole-body scanner (Philips Medical Systems, Best, The Netherlands) and an in-house ^13^C/^1^H surface coil (12 cm diameter ^13^C coil; Fig. 2B) positioned over the liver. A representative image showing positioning of the ^13^C coil is shown in Fig. 2D,F. ^13^C spectra were acquired using a pulse-acquired sequence with ^1^H WALTZ decoupling (TR = 1.5 seconds; nominal tip angle = 90 degrees; 1,024 data points; 8-kHz SW; 15-minute scan duration). Concentrations of ^13^C-labeled metabolites were determined from spectra as described in the Supporting Materials.

## Results

### Preclinical Studies: Perturbed GSH Turnover Is Observed After Acute and Chronic OS Insults

Table 1 shows rat morphometric measurements, plasma biochemical analysis, and hepatic F_2_-isoprostane content analysis. CCl_4_ treatment resulted in a significant elevation of plasma aspartate aminotransferase (AST) and alanine aminotransferase (ALT), relative to the control group, indicative of acute liver damage (*P* < 0.05). Quantitation of hepatic F_2_-isoprostanes as a biomarker of LPO demonstrated significant elevation in both the CCl_4_-treated and HFHC diet groups, compared to control, indicating OS insults.

**Table 1:** Preclinical Study Morphometric Measurements, Plasma Biochemical Analysis, and Hepatic Isoprostane Content Analysis

						Hepatic Isoprostane Content (ng/g)		
	Body Mass (g)	Liver Mass (g)	ALT (U/L)	AST (U/L)	ALP (U/L)	F_2_-III	F_2_-IV	F_2_-VI
Control	220 ± 33	10.8 ± 0.8	49 ± 8	71 ± 6	290 ± 17	1.4 ± 0.3	0.06 ± 0.19	1.7 ± 0.2
CCl4	243 ± 27	11.6 ± 0.8	378 ± 84	950 ± 378	281 ± 27	46.7 ± 10.9	0.90 ± 0.19	36.4 ± 3.2
HFHC diet	527 ± 67	17.0 ± 3.1	74 ± 34	200 ± 140	136 ± 43	20.2 ± 2.8	0.60 ± 0.14	5.7 ± 1.3

Abbreviation: ALP, alkaline phosphatase.

**Fig 3:**
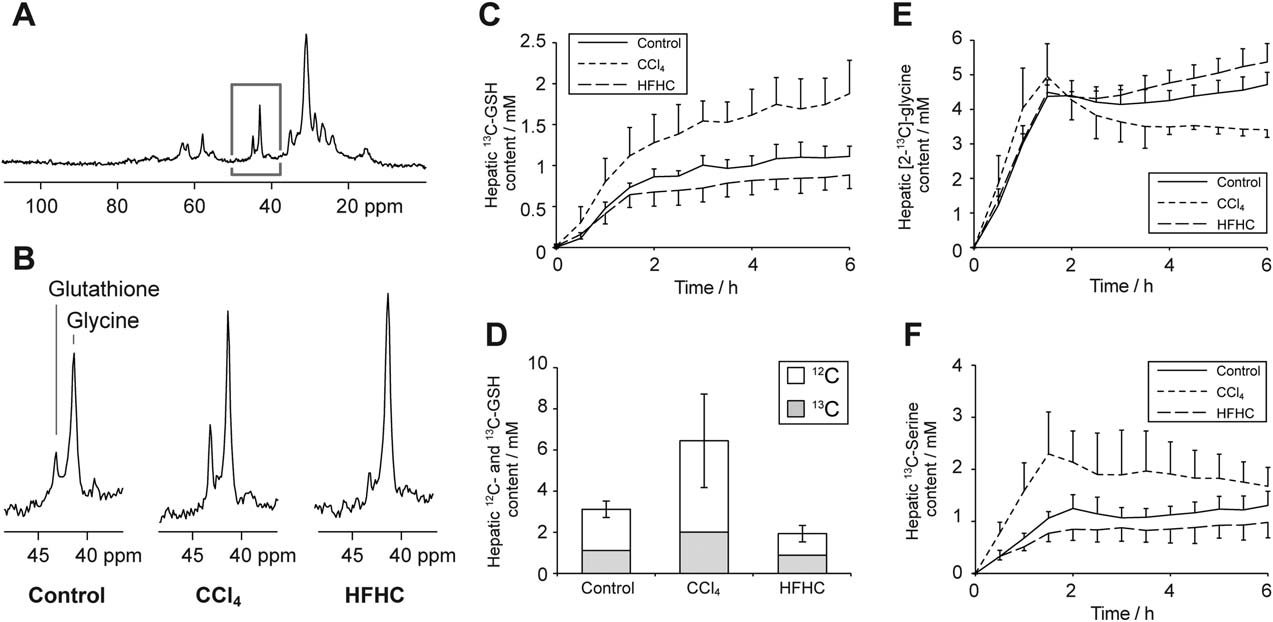
Preclinical study ^13^C MR data. (A) Representative hepatic ^13^C spectrum acquired after 6 hours of [2-^13^C]-glycine infusion. (B) Regions of the ^13^C spectrum showing resonances from [2-^13^C]-glycine and ^13^C-labeled GSH from rats in each of the three study groups. (C) Time course of hepatic ^13^C-labeled GSH concentration in the experimental groups. (D) Mean concentrations of ^13^C-labeled and total GSH in the experimental groups at the end of the 6.5-hour experiment. (E and F) Time courses of hepatic [2-^13^C]-glycine and [2-^13^C]-serine concentration in the experimental groups.

Figure 3 shows ^13^C spectra and calculated metabolite concentrations from the preclinical experimental studies. Figure 3A shows a representative rat hepatic ^13^C spectrum acquired at 6 hours after commencement of [2-^13^C]-glycine administration. Resonances from infused [2-^13^C]-glycine and endogenously produced ^13^C-labeled GSH are clearly observed at 42.4 and 44.2 parts per million (ppm), respectively, adjacent to natural-abundance ^13^C resonances from lipids in the 35-14-ppm region. The highlighted region of this spectrum (50-40 ppm) is shown in Fig. 3B, comprising representative spectra from the three experimental groups acquired at the 5-hour experimental time point, showing the [2-^13^C]-glycine and ^13^C-GSH resonances. Differences in both ^13^C-GSH and [2-^13^C]-glycine concentration are apparent between the groups, reflecting insult-induced differences in both GSH and glycine metabolism.

Dynamic measurements of hepatic ^13^C-labeled GSH content were made over the experimental time course and are shown, for the three groups, in Fig. 3C. A marked, statistically significant (*P* < 0.05) elevation of ^13^C-GSH content was observed in the CCl_4_-treated group, compared to the other groups (1.71 ± 0.39, compared to 1.11 ± 0.19, mmol/L for the control group at 5 hours), consistent with hepatic response to the acute OS insult. Conversely, rats in the HFHC diet group showed slightly lower ^13^C-GSH than the control group (0.86 ± 0.18 mmol/L), indicating a lower GSH turnover, though this difference was not statistically significant. However, total GSH content was significantly lower than control in the HFHC group, and significantly elevated in the CCl_4_-treated group (*P* < 0.05; Fig. 3D). The data demonstrate the model of early NASH exhibiting compromised OS defenses (with consequent potential for cellular damage), in contrast to the acute CCl_4_ insult, resulting in a strong up-regulation of GSH production. The rate of ^13^C-label incorporation into the hepatic GSH pool also reflects these group differences, showing a higher rate for CCl_4_-treated rats than for the other groups, with an initial rate (measured between t = 30 and 90 minutes) of 0.76 ± 0.14 mmoles/kg/hour, compared to 0.58 ± 0.09 and 0.48 ± 0.07 mmoles/kg/hour for the control and HFHC groups, respectively (*P* < 0.05).

Hepatic [2-^13^C]-glycine content over the experiment duration is shown in Fig. 3E, charting the effect of label infusion. All three groups show a similar pattern: a rapid rise during the initial 90-minute high infusion rate, followed by maintenance of [2-^13^C]-glycine concentration at approximately 4 mmol/L. The infusion scheme was chosen to rapidly maximize and then maintain hepatic glycine ^13^C fractional enrichment. Minor differences between groups are observed after approximately 3 hours; the CCl_4_-treated group shows a downward trend in [2-^13^C]-glycine concentration from 2 to 6 hours, whereas the HFHC diet group shows a slight upward trend. ^1^H MRS measurements of glycine ^13^C fractional enrichment in extract samples were 57% ± 4%, 59% ± 5%, and 69% ± 2% in the control, CCl_4_-treated, and HFHC groups, respectively, at the end of the infusion period, with the HFHC group measurement significantly different from control (*P* < 0.05). We attribute these differences to variations in flux through the glycine metabolism pathways: In addition to alterations in flux of ^13^C label to GSH, we also observed differences in flux through serine hydroxymethyltransferase (SHMT) to ^13^C-labeled serine. Our preclinical study protocol did not permit serial sampling of plasma to measure glycine ^13^C fractional enrichment, but previous studies by Macdonald et al.^11^ and by Fern and Garlick^17^ demonstrate that a similar infusion of labeled glycine results in steady state being achieved rapidly and within 2 hours, and our human studies demonstrated glycine fractional enrichment reaching steady state within 2 hours (*vide infra*).

Figure 3F shows hepatic [2-^13^C]-serine content, with the CCl_4_-treated group showing an elevated concentration of labeled serine, compared to the other groups. This difference was statistically significantly, compared to control, at the 1.5-hour time point and compared to the HFHC group at the 1.5-, 3-, and 5-hour time points (*P* < 0.05).

**Fig 4:**
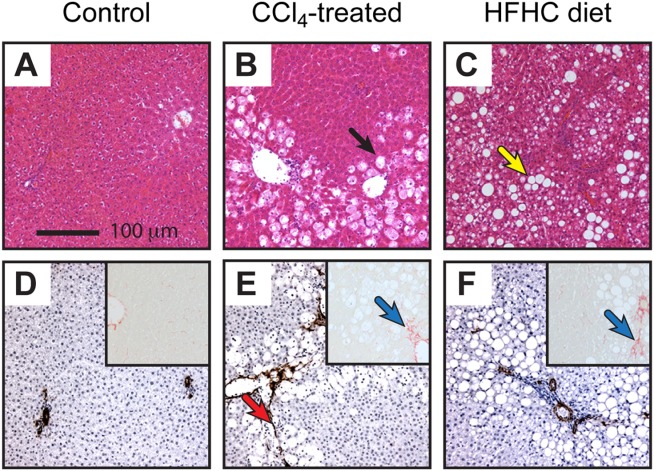
Histological sections of rat liver from the preclinical study groups. (A-C) H&E-stained sections. (D-F) α-SMA stained sections and Sirius Red–stained sections (inset). Representative photomicrographs at 100× magnification of a minimum of n = 3 rats per group. Black arrow indicates damaged hepatocytes, yellow arrow indicates fat deposition, red arrow indicates α-SMA staining of myofibroblasts, and blue arrows indicate collagen deposition.

Figure 4 shows histological sections from the three groups stained with hematoxylin and eosin (H&E), alpha-smooth muscle actin (α-SMA) for myofibroblast visualisation, and Sirius Red as a marker of collagen deposition. Liver histology was normal for the control group, whereas the CCl_4_ treatment group showed hallmarks of acute OS (ballooned and swollen dead or dying hepatocytes and the emergence of hepatic myofibroblasts). The HFHC group showed early steatohepatitis, including fat deposition, activation of hepatic myofibroblasts, and collagen deposition.

MS analysis of GSH tissue extracts identified a fraction of the GSH containing two ^13^C nuclei per molecule. We have observed similar labeling in our previous studies of tumour OS defenses^13^ and attribute this to synthesis of ^13^C-label in the cysteinyl residue of GSH. Incorporation of ^13^C label from [2-^13^C]-glycine into cysteine occurs through the trans-sulfuration pathway,^18^ with labeled serine as an intermediate. The percentage of doubly ^13^C-labeled GSH was 2.9% ± 0.8%, 4.1% ± 1.7%, and 6.2% ± 1.6% for the control, CCl_4_-treated, and HFHC diet groups, respectively. The HFHC group showed a significantly higher degree of double ^13^C-labeling than the other groups (*P* < 0.05), indicating higher production of cysteine through the trans-sulfuration pathway. This may originate from dietary differences between groups, with lower protein intake in the HFHC group resulting in up-regulation of trans-sulfuration to supply cysteine.^18^

### Human Studies: Hepatic ^13^C-Labeled GSH Can Be Detected and Quantified After Ingestion of [2-^13^C]-Glycine

**Fig 5:**
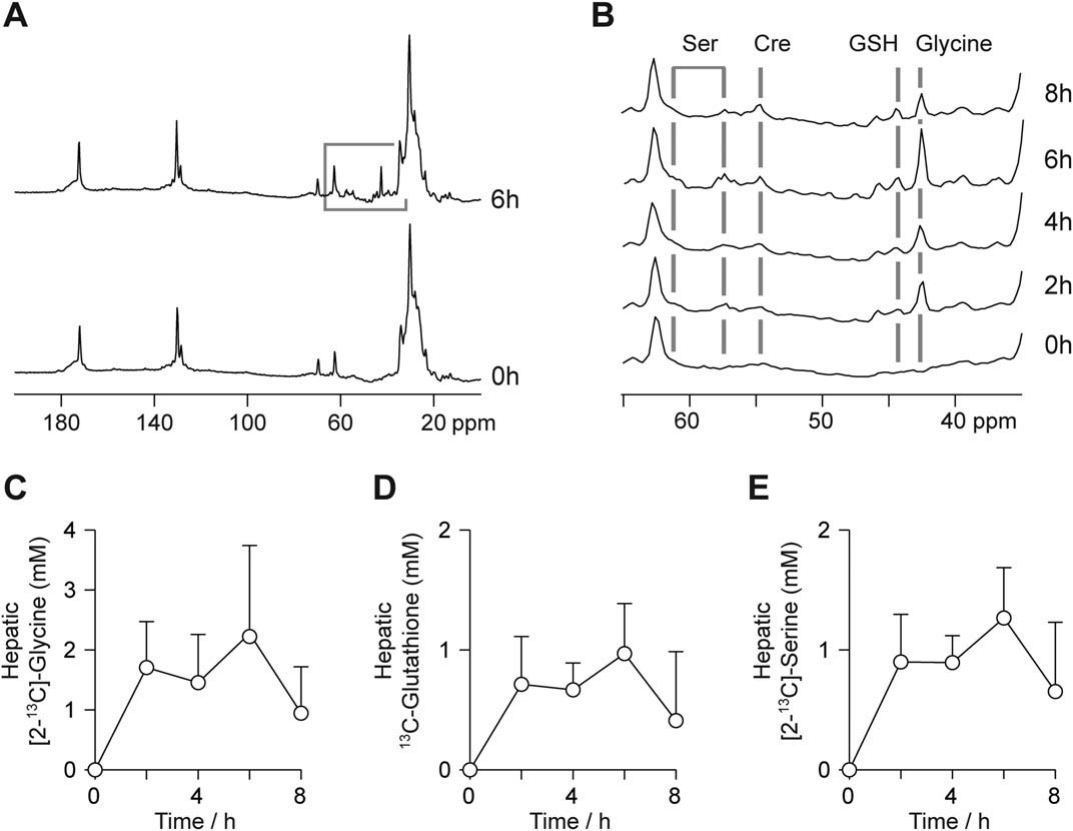
Human study ^13^C MR data. (A) Hepatic ^13^C spectra from a study volunteer before and after the 6-hour glycine ingestion period. (B) Region of the ^13^C spectrum from a study volunteer showing resonances from [2-^13^C]-glycine and endogenously ^13^C-labeled GSH, serine, and creatine. (C-E) Mean concentrations of [2-^13^C]-glycine, ^13^C-labeled GSH, and [2-^13^C]-serine over the experimental time course.

Study participants were male and had an average age of 32 ± 4 years and a body weight of 75 ± 3 kg. Standard plasma clinical biochemistry assays reported normal results for all participants. Hepatic ^13^C spectra showed well-resolved resonances from [2-^13^C]-glycine and ^13^C-GSH, allowing quantification of ^13^C-label incorporation into GSH over the experimental time course. Figure 5A shows spectra from a participant acquired before and at 6 hours after commencement of [2-^13^C]-glycine ingestion. The spectrum was similar in appearance to the preclinical studies (reflecting the similarities in experimental approach), though a broader region of the spectrum was observed as a result of the lower magnetic field strength of the human whole-body scanner (3T, rather than 7T). Figure 5B shows the 65-35-ppm spectra region from the same subject at the five experimental time points, showing the administered [2-^13^C]-glycine and its subsequent metabolism. Signal from labeled glycine was observed at the first measurement after ingestion (2 hour), and its magnitude was maximal at the end of the 6-hour ingestion period. Incorporation of the ^13^C label into the 2-carbon of the glycinyl residue of GSH (44.2 ppm) and into other metabolites of glycine was also observed from the 2-hour time point onward. These other metabolites included [2-^13^C]- and [3-^13^C]-serine at 57.4 and 61.3 ppm, respectively, choline and/or creatine at 54.8 ppm, and a resonance at 45.8 ppm that may originate from intermediates in the formation of creatine and choline, guanidoacetate, or dimethylglycine. Figure 5C-E shows the mean concentrations of ^13^C-labeled glycine, GSH, and serine in human liver over the experimental time course. The data demonstrate the rapid turnover of GSH, and rapid flux of glycine to serine, with the appearance of both by the 2-hour time point and appreciable drop in concentration 2 hours after ingestion of [2-^13^C]-glycine had ceased (8-hour time point).

**Fig 6:**
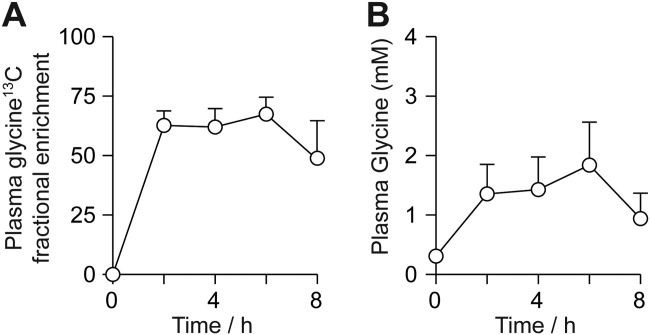
Human study plasma glycine analysis. (A) Mean ^13^C fractional enrichment of plasma glycine. (B) Mean glycine (unlabeled plus labeled) concentration over the experimental time course.

Figure 6 shows the ^13^C fractional enrichment and total concentration of plasma glycine in the human studies. ^13^C fractional enrichment was constant at 64.0% ± 6.6% during the glycine ingestion period. This indicates that steady-state labeling was achieved, and thus the incorporation of ^13^C label into GSH reflects the flux through the GSH pathway, rather than the changing ^13^C-labeling of the glycine pool. The concentration of plasma glycine peaked at 1.8 ± 0.7 mM at the 6-hour time point.

The initial rate of incorporation of ^13^C label into GSH, measured between the 0- and 2-hour time points, was 0.32 ± 0.18 mmol/kg/hour. The highest concentrations of ^13^C-labeled metabolites were observed at the end of the [2-^13^C]-glycine administration period; peak hepatic [2-^13^C]-glycine concentration was 2.3 ± 1.5 mmol/L, whereas ^13^C-GSH and [2-^13^C]-serine concentrations were 1.0 ± 0.4 and 1.3 ± 0.6 mmol/L, respectively.

## Discussion

### ^13^C MRS Provides a Repeatable Noninvasive *In Vivo* Measure of GSH Synthetic Rate

Our data demonstrate successful quantitation of endogenously produced ^13^C-labeled GSH in human liver and perturbed GSH concentration and turnover rate in rodent models of acute and chronic hepatic OS. The noninvasive nature of MR provides a safe, repeatable tool applicable to hepatic clinical research, avoiding the need for biopsy sampling. A broad range of biomarkers have been employed in numerous preclinical and clinical studies to report on OS. Those centred on GSH include GSH content, activities of enzymes involved in GSH synthesis and adduct formation, and concentrations of other cellular antioxidants and activities of their associated enzymes.^5^,^19^–^22^ However, GSH concentration and enzyme activity measurements do not necessarily reflect flux through GSH synthesis and utilization pathways, and small differences in the balance between GSH synthesis and utilization may underlie the literature conflict on, for example, whether CCl_4_ causes an acute increase or decrease in GSH content.^4^,^22^,^23^ In this regard, our dynamic ^13^C-labeling approach may afford a useful, robust metric for assessment of cellular response to hepatic OS by reporting on flux, rather than steady-state concentrations of GSH.

Although our method does not distinguish between reduced and oxidized GSH (because the chemical shift of the ^13^C label is unaffected by GSH redox state), our data demonstrate that changes in hepatic GSH turnover and content provide a sensitive redox defense biomarker. Future work using a labeling scheme of a ^13^C nucleus close to the GSH thiol group may allow the reduced and oxidized forms of GSH to be distinguished. The present studies were conducted using [2-^13^C]-glycine because this compound is well tolerated at elevated concentrations, having a ^13^C resonance distinct from other resonances in the spectrum and, unlike cysteine, is not rate limiting for GSH synthesis.^5^ A method to measure GSH synthesis by incorporation of ^3^H-labeled cysteine into GSH has been previously employed in clinical research studies.^24^,^25^ Our method has some similarities in approach, but has the advantage of providing a direct, dynamic, and radiolabel-free tissue GSH content measurement.

Performance of ^13^C MRS is not a standard capability for the majority of MRI scanners in current use. However, all major MRI scanner manufacturers have 3T scanner products capable of ^13^C spectroscopy, and appropriate radiofrequency (RF) coils are commercially available. We consider our technique most suited to clinical research studies at present (where human application of ^13^C MRS has played an important role to date), and precedents exist for translation of ^13^C MR to use in clinical diagnosis.^26^ The cost of [2-^13^C]-glycine for our studies was twice the cost of per-volunteer access to the MR scanner and thus not prohibitive for clinical research. Furthermore, refinement of the experimental protocol to use lower quantities of ^13^C label (by lower total dose or shorter administration period) may be possible given the achieved spectral quality. The higher variance in label incorporation into GSH in humans, compared with the preclinical studies, may reflect greater interindividual biological variation and/or differences in methodology (oral vs. IV label administration). However, the variance in human ^13^C-GSH synthesis rates was similar to that observed in other studies with similar methodological approaches, such as measurement of human hepatic glycogen synthesis rate, as reported on previously.^27^

Brain glutathione content measurements have been made by ^1^H MRS.^28^,^29^ This approach is well suited to normal and neoplastic brain tissue, but is unable to report on GSH flux and performs less well in other tissues as a result of shorter T_2_ relaxation times and significant contributions to the ^1^H spectrum from lipids. A recent and novel MR approach has employed dynamic nuclear polarization (DNP) methods to measure the equilibrium flux between ^13^C-labeled ascorbic and dehydroascorbic acid as an *in vivo* probe of tissue redox state.^30^,^31^ DNP provides a method to greatly increase the MR signal from an exogenously administered compound, such as ^13^C-labeled ascorbate, and permits measurement of enzyme flux over a short (tens of seconds) time scale. However, the approach requires administration of considerable quantities of ^13^C-labeled tracer, which may have the potential to alter redox balance. In our studies, we employed conventional ^13^C spectroscopy, permitting measurements of GSH metabolism over a 6.5-hour time period and using a tracer molecule that can be administered without altering the rate of GSH synthesis.^5^

### Hepatic OS Results in Significant Perturbation of GSH Turnover, Measurable by Noninvasive ^13^C MRS

Our human studies represent the translation of a novel methodology with potential for direct quantitation of GSH content and synthesis rate in humans as a biomarker. Studies of biopsy samples have demonstrated decreased GSH content in nonalcoholic fatty liver disease (NAFLD) and NASH patients^10^,^32^ (a finding echoed by our preclinical data) and have investigated the effect of therapeutic regimes.^33^ Our noninvasive approach provides a direct measurement from liver tissue (as compared with indirect blood biomarkers) without the need for invasive biopsy. The preclinical models of acute and chronic OS exhibited insult impact in tissue histology, plasma biomarker assay results, MS analysis of isoprostane content, and in ^13^C MR measures of glycine and GSH metabolism. A major acute OS insult with adaptive changes in GSH concentration and turnover was produced by CCl_4_ administration, whereas a mild chronic insult with evidence of compromised OS defenses and early histological signs of steatohepatitis was produced in the HFHC dietary model. F_2_-isoprostanes provide a biomarker of LPO and thus tissue OS,^34^ and elevated concentrations have been reported in patients with NAFLD^35^ and in rat models of hepatic fibrosis.^36^ The elevated levels observed in the CCl_4_-treated and HFHC diet groups provides confirmation that the experimental insults resulted in hepatic OS.

The CCl_4_-treated group exhibited an approximately 50% increase in GSH concentration and ^13^C label incorporation rate, reflecting a cellular response of increased GSH synthesis and utilization. We chose to perform our MR studies at 12 hours after CCl_4_ administration to coincide with a large impact on tissue OS and cellular response to the insult,^37^ and the significant effect of the insult was also observed in the biochemical and histological measurements.

Previous studies using the HFHC dietary model of NASH have demonstrated florid NASH developing over a 16-week period.^15^ Our studies employed an 8-week HFHC dietary feeding period, and isoprostane analysis and histology confirmed hepatic OS and the appearance of a NASH phenotype in our experimental group at the end of this period. Our studies were limited to an 8-week duration, in part, by the animal-size constraints imposed by our preclinical MR scanner. Decreased hepatic GSH content was observed in this group, along with elevated flux through the trans-sulfuration pathway and decreased SHMT flux. Future studies to investigate the progression of the HFHC dietary model to florid NASH beyond the stage employed in our studies would provide information on the changing hepatic OS environment and resultant cellular adaptations.

In summary, our data demonstrate *in vivo* ^13^C-labeling and noninvasive detection of GSH after ingestion (human) or IV infusion (rat) of [2-^13^C]-glycine. Clinical research applications for this novel methodology include directly quantifying the effect of GSH-depleting xenobiotics, such as acetaminophen, drugs that elevate GSH synthesis rate by supply of rate-limiting substrate, such as *N*-acetyl cysteine or *S*-adenosyl methionine, and of disease processes involving OS and perturbed hepatic OS defences, such as steatohepatitis.

## Abbreviations

ALT: alanine aminotransferase
AST: aspartate aminotransferase
DNP: dynamic nuclear polarization
^13^C-glutathione: γ-glutamylcysteinyl-[2-^13^C]-glycine
GSH: glutathione
H&E: hematoxylin and eosin
HFHC: high-fat, high-carbohydrate diet
IP: intraperitoneal
IV: intravenous
LPO: lipid peroxidation
MRI: magnetic resonance imaging
MRS: magnetic resonance spectroscopy
MS: mass spectrometry
NAFLD: nonalcoholic fatty liver disease
NASH: nonalcoholic steatohepatitis
^1^H NMR: proton nuclear magnetic resonance
OS: oxidative stress
ppm: parts per million
RF: radiofrequency
ROS: reactive oxygen species
SHMT: serine hydroxymethyltransferase
α-SMA: alpha-smooth muscle actin
