# Supplementary material for: Noninvasive in vivo magnetic resonance measures of glutathione synthesis in human and rat liver as an oxidative stress biomarker
Source: Hepatology. 2014 Apr 25;59(6):2321–30. doi: 10.1002/hep.26925 (PMC4160151; doi:10.1002/hep.26925)
Supplement: Supplementary file 1 [file hep0059-2321-SD1.pdf]

Supplementary Material: Methods

Human study: Scanner RF power and B_0 field optimisation for ^{13}C MR spectroscopy

RF power requirements were optimised for each subject prior to acquisition of study data to ensure consistency of ^{13}C tip angle and decoupler efficacy. ^{13}C calibration data were acquired from a fiducial marker within the coil housing that exhibited a ^{13}C signal with short T_1 (213 mM [2-^{13}C]-acetone and 25 mM $GdCl_3$ in water). ^{13}C spectra were acquired using a pulse-acquire sequence at nominal tip angles of 25, 50, 100 and 200° (repetition time = 150 ms, spectral width = 8 kHz, 256 datapoints, 250 averages), and the discrepancy between requested and achieved tip angle calculated from the variation in signal intensity between the four spectra. This calibration procedure was designed to enable correction of differences in achieved ^{13}C tip angle between study volunteers resultant from differences in coil loading. However, in practice the procedure demonstrated that all three volunteers and the calibration phantom had the same ^{13}C RF power requirements, reflecting the similarity in coil loading between scans.

Consistency of ^{1}H decoupler power was also optimised for study scans. Tests performed during coil development demonstrated consistent decoupler efficacy over the active volume of the ^{13}C coil above a threshold power value, and we have successfully employed acquisition of ^{1}H-decouped ^{13}C spectra in previous studies (1-3). In coil development studies we observed that automated scanner ^{1}H power calibration preparation steps performed prior to scan execution have the potential to introduce a variation in ^{1}H RF power output when scans were acquired using our ^{13}C/^{1}H surface coil. We attributed this to the ^{1}H power calibration procedure being optimised for homogeneous B_1 fields generated by the scanner's body coil rather than the inhomogeneous B_1 field of a surface coil. Thus automated ^{1}H power optimisation preparation steps were disabled for this study, and consistency of ^{1}H power output monitored via the scanner ^{1}H RF amplifier power meter (standard deviation of decoupler power = ± 1.7%). Efficacy of ^{1}H decoupling over the active volume of the ^{13}C coil was confirmed in phantom studies employing ^{1}H-decoupled ^{13}C 1D chemical shift imaging.

Prior to acquisition of ^{13}C spectra, homogeneity of the scanner's B_0 magnetic field was optimised over a 16 x 16 x 12 cm^3 volume of interest located over the subject's liver using a B_0-mapping method implemented by the scanner manufacturer, based on the method described by Schär et al (4), which calculates optimal scanner shims from water proton frequency measurements acquired from dual echo multislice gradient echo images covering the volume of interest.

Preclinical study: Scanner RF power and B_0 field optimisation for ^{13}C MR spectroscopy

^{13}C and ^{1}H power requirements for the RF coils were calibrated a similar approach to the human studies, employing a fiducial marker within the coil housing as a signal source for ^{13}C power calibration, and a volume-selective spectroscopy scan localised to the rat liver for ^{1}H power calibration.

Homogeneity of the scanner's B_0 magnetic field was performed by iterative manual adjustment of shim currents with concurrent measurement of water proton resonance linewidth from a 2 x 2 x 1 cm^3 volume of interest positioned over the liver. ^{1}H linewidth was 97 ± 19 Hz (mean ± standard deviation) for the preclinical studies.

Relative contributions of hepatic and non-hepatic tissue to ^{13}C spectra

A surface coil was used to localise the acquired spectra to liver tissue in our study. Prior to human studies we performed measurements to determine the relative contribution of hepatic and non-hepatic tissue to the acquired signal. The active volume of the coil is principally liver, but also contains skin, subcutaneous fat, bone, and intercostal muscle. As the magnitude of the ^{13}C coil's B_1 field decreases with distance from the coil, a pulse power setting optimised for maximal hepatic signal results in an achieved tip angle greater than 90 degrees between the coil and the liver. This results in T_1-saturation immediately adjacent to the coil, with consequent reduction of the contribution of non-hepatic regions to the acquired spectrum. Based on our coil sensitivity measurements, on the morphology of study participants, and on the acquisition parameters used in the scan protocol, we estimate that hepatic tissue comprises 86 ± 4% by volume to the study spectra (ie. if a metabolite were distributed uniformly over hepatic and non-hepatic regions, 86% of the signal would arise from the liver (data not shown)). As non-hepatic tissue has lower glutathione content than liver, this further increases the dominance of the hepatic contribution. For these

reasons we consider the contributions to the spectra from non-hepatic tissue to be sufficiently minor that they can be neglected in this study.

Acquisition of preclinical ^{13}C spectra to present a similar situation, aided further by the more ventral morphology of the rat liver (allowing coil positioning with minimal contribution from the ribcage and minimal separation between coil and liver). Little subcutaneous fat was observed adjacent to the coil, even in rats receiving the HFHC diet, and imaging prior to acquisition of ^{13}C spectra confirmed consistent positioning of the liver over the ^{13}C RF coil (Figure 1 F&H). Thus for preclinical studies the contributions of non-hepatic tissue to the spectra were assumed to be negligible.

Human studies: data analysis and quantitation

Experimental ^{13}C spectral datasets were Fourier transformed after application of 15 Hz exponential line broadening, then manually phased. Peak integrals were determined for resonances originating from [2-^{13}C]-glycine (42.4 ppm), ^{13}C-glutathione (44.2 ppm), [2-^{13}C]- and [3-^{13}C]-serine (57.4 and 61.3 ppm respectively), and choline and/or creatine (54.8 ppm). The initial processing of spectra was performed using jMRUI version 4.0 (Universitat Autònoma de Barcelona, Spain) (5,6), and calculation of peak magnitude was performed using Matlab (The Mathworks, Natick, MA, USA) by spectral baseline correction followed by integration of regions of the spectrum corresponding to the peaks of interest.

^{13}C spectra were also acquired from a liver shaped phantom filled with a solution of 934 mM glycine (containing 10 mM [2-^{13}C]-glycine at ^{13}C natural abundance) and 100 mM potassium chloride, and the magnitude of the [2-^{13}C]-glycine resonance used as a concentration standard for quantitation of in vivo data. These spectra were acquired with the phantom positioned at a separation from the RF coil equal to the separation between coil and liver in the in vivo studies, and ^{1}H-decoupled ^{13}C spectra were acquired with the same scan acquisition parameters as used for the in vivo human studies. The concentration of potassium chloride in the phantom was chosen to produce coil loading equivalent to the in vivo situation, as measured by ^{13}C coil power requirements as described above. Quantitation of data in this manner assume that [2-^{13}C]-glycine in the phantom has the same T_1 as occurs in vivo. Statistical significance of differences in concentrations and rates between experimental groups was determined using one-way ANOVA with Tukey multiple comparisons (Minitab 16, Minitab Inc, State College, PA, USA).

Preclinical studies: data analysis and quantitation

Analysis of rat MR data was performed using jMRUI version 4.0 (Universitat Autònoma de Barcelona, Spain) (5,6). Spectral datasets were zero-filled from 512 to 1024 datapoints, 15 Hz of exponential line broadening was applied, then data were Fourier transformed and manually phased. The AMARES fitting algorithm (7) was employed to determine the magnitude of resonances arising from [2-^{13}C]-glycine (42.4 ppm), ^{13}C-glutathione (44.2 ppm), [2-^{13}C]- and [3-^{13}C]-serine (57.4 and 61.3 ppm respectively), and choline and/or creatine (54.8 ppm).

As with human studies, in vivo ^{13}C metabolite concentrations were determined by comparing fitted ^{13}C peak areas from in vivo studies to the magnitude of the [2-^{13}C] resonance acquired from a liver-shaped phantom containing a solution of 5mM [2-^{13}C]-glycine and 100 mM potassium chloride. Again, the positioning and coil loading of the phantom studies replicated the in vivo experiment.

Statistical significance of differences in concentrations and rates between experimental groups was determined using one-way ANOVA with Tukey multiple comparisons (Minitab 16, Minitab Inc, State College, PA, USA).

Human study: Analysis of plasma glycine content and ^{13}C fractional enrichment

Perchloric acid extracts were prepared from plasma samples and extracts analysed by ^{1}H MR spectroscopy to determine the concentration of unlabelled and [2-^{13}C]-labelled glycine. 50uL of 5M perchloric acid was added to 0.75 mL aliquots of plasma and the solution thoroughly mixed. Samples were centrifuged at 16,000 x g for 5 minutes to remove precipitated components, and 50uL of 1M sodium phosphate solution added to the supernatant as a pH buffer. The resultant solution was neutralised with 1.25M potassium hydroxide solution, centrifuged at 16,000 x g for 5 minutes, then the supernatant freeze dried and resuspended in 0.5 mL D_2O. 5 uL of 100 mM trimethylsilyl propionate in D_2O was added as a chemical shift and concentration reference compound. ^{1}H pulse-acquire spectra were acquired from the samples using Bruker Avance III 500 MHz spectrometer equipped with a BBO probe. The data were Fourier Transformed with 2 Hz exponential line broadening and integral determined for resonances from protons at the C2 position of [2-^{12}C]- and [2-^{13}C]-glycine at 3.57 and 3.71 ppm respectively. Concentrations of labelled and unlabelled glycine were determined by comparison of peak integrals to those acquired

from a standard solution containing 1 mM [2-^{12}C]-glycine and 2 mM [2-^{13}C]-glycine that had been prepared using the same protocol as for plasma samples.

Preclinical study methods: Preparation and analysis of tissue extracts

Frozen liver samples were homogenized at room temperature in preweighed solutions of 50 mM phosphate buffer containing 25 mM monobromobimane. Proteins were precipitated by addition of perchloric acid and removed by centrifugation, then excess perchloric acid was removed by neutralisation with potassium hydroxide. The resultant thiol-bimane conjugate extracts were freeze dried for storage, then analysed by mass spectrometry using a Thermo Surveyor liquid chromatograph coupled to a Thermo LTQ linear ion trap mass spectrometer. The fractional enrichment of glutathione-bimane with ^{13}C label was determined as previously described (8). Briefly, the isotope enrichment was calculated from the ratio of distributions of mass to charge ratio of 500, 499 and 498 amu by comparison to theoretical isotope distribution patterns calculated for glutathione-bimane using a mass spectrometry webtool (9). Glutathione concentration in the extracts was determined using HPLC analysis of glutathione-bimane conjugates. A portion of tissue extract samples was freeze dried, resuspended in D_2O, and ^{1}H MR spectra acquired using a Bruker Avance III 500 MHz spectrometer equipped with a BBO probe for n=3 samples per experimental group.

Hepatic F_2-isoprostane were isolated from liver tissue using the methods of Davies (10) and Morrow (11), comprising chloroform/methanol extraction, hydrolysis of esterified isoprostanes in phospholipids, and purification by cation exchange solid phase extraction. F_2-isoprostane analysis was performed using a Prominence HPLC (Shimadzu, Kyoto, Japan) equipped with a C_{18} column, coupled to a Q-Trap mass spectrometer (Sciex, Warrington, UK) operated using multiple reaction monitoring in negative ion mode, monitoring the transition of the precursor F_2 isoprostane ion (with a m/z of 353) to specific productions with m/z of 193, 127 and 115 for the F_2-III, IV and VI isomers respectively.

Preclinical study: Histology

Formalin fixed liver sections were stained with Haematoxyin and Eosin (H&E) or 0.1% Sirius Red Picric solution following standard protocols. α-smooth muscle actin

antibody immunohistochemistry (α-SMA) was performed on formalin-fixed liver sections as previously described (12). Photomicrographs were taken at x10 magnification on a Leica DMR microscope with a DFC 310 FX camera (Leica Microsystems, Wetzlar, Germany).

References

1. Lim EL, Hollingsworth KG, Smith FE, Thelwall PE, Taylor R. Effects of raising muscle glycogen synthesis rate on skeletal muscle ATP turnover rate in type 2 diabetes. Am. J. Physiol. Endocrinol. Metab. 2011;

2. Lim EL, Hollingsworth KG, Smith FE, Thelwall PE, Taylor R. Inhibition of lipolysis in Type 2 diabetes normalizes glucose disposal without change in muscle glycogen synthesis rates. Clin. Sci. 2011;121:169–177.

3. Stevenson EJ, Thelwall PE, Thomas K, Smith F, Brand-Miller J, Trenell MI. Dietary glycemic index influences lipid oxidation but not muscle or liver glycogen oxidation during exercise. Am. J. Physiol. Endocrinol. Metab. 2009;296:E1140–7.

4. Schär M, Kozerke S, Fischer SE, Boesiger P. Cardiac SSFP imaging at 3 Tesla. Magn Reson Med. 2004;51:799–806.

5. Naressi A, Couturier C, Devos JM, Janssen M, Mangeat C, de Beer R, et al. Java-based graphical user interface for the MRUI quantitation package. MAGMA. 2001;12:141–152.

6. Naressi A, Couturier C, Castang I. ScienceDirect.com - Computers in Biology and Medicine - Java-based graphical user interface for MRUI, a software package for quantitation of in vivo/medical magnetic resonance spectroscopy signals. Computers in biology and medicine. 2001;31:269–286.

7. Vanhamme L, Van Huffel S, Van Hecke P, van Ormondt D. Time-domain quantification of series of biomedical magnetic resonance spectroscopy signals. J. Magn. Reson. 1999;140:120–130.

8. Gamcsik MP, Dubay GR, Cox BR. Increased rate of glutathione synthesis from cystine in drug-resistant MCF-7 cells. Biochem. Pharmacol. 2002;63:843–851.

9. Manura JJ, Manura DJ, editors. Isotope Distribution Calculator and Mass Spec Plotter. Scientific Instrument Services; [cited 2013 Jan 9]. Available from: http://www.sisweb.com/mstools/isotope.htm

10. Davies SS, Amarnath V, Brame CJ, Boutaud O, Roberts LJ. Measurement of chronic oxidative and inflammatory stress by quantification of isoketal/levuglandin gamma-ketoaldehyde protein adducts using liquid chromatography tandem mass spectrometry. Nat Protoc. 2007;2:2079–2091.

11. Zeiger SLH, Musiek ES, Zanoni G, Vidari G, Morrow JD, Milne GJ, et al. Neurotoxic lipid peroxidation species formed by ischemic stroke increase injury. Free Radic. Biol. Med. 2009;47:1422–1431.

12. Oakley F, Mann J, Nailard S, Smart DE, Mungalsingh N, Constandinou C, et al. Nuclear factor-kappaB1 (p50) limits the inflammatory and fibrogenic responses to chronic injury. Am. J. Pathol. 2005;166:695–708.
